# Systematic Investigation of Resistance Evolution to Common Antibiotics Reveals Conserved Collateral Responses across Common Human Pathogens

**DOI:** 10.1128/AAC.01273-20

**Published:** 2020-12-16

**Authors:** Mari C. Rodriguez de Evgrafov, Marius Faza, Konstantinos Asimakopoulos, Morten O. A. Sommer

**Affiliations:** a The Novo Nordisk Foundation Center for Biosustainability, Technical University of Denmark, Lyngby, Denmark

**Keywords:** human pathogens, resistance evolution, antibiotic resistance, collateral sensitivity, cross-resistance, drug resistance evolution

## Abstract

As drug resistance continues to grow, treatment strategies that turn resistance into a disadvantage for the organism will be increasingly relied upon to treat infections and to lower the rate of multidrug resistance. The majority of work in this area has investigated how resistance evolution toward a single antibiotic effects a specific organism’s collateral response to a wide variety of antibiotics. The results of these studies have been used to identify networks of drugs which can be used to drive resistance in a particular direction. However, little is known about the extent of evolutionary conservation of these responses across species. We sought to address this knowledge gap by performing a systematic resistance evolution study of the ESKAPE pathogens (Enterococcus faecium, Staphylococcus aureus, Klebsiella pneumoniae, Acinetobacter baumannii, Pseudomonas aeruginosa, and Enterobacter cloacae) under uniform growth conditions using five clinically relevant antibiotics with diverse modes of action. Evolved lineages were analyzed for collateral effects and the molecular mechanisms behind the observed phenotypes. Fourteen universal cross-resistance and two global collateral sensitivity relationships were found among the lineages. Genomic analyses revealed drug-dependent divergent and conserved evolutionary trajectories among the pathogens. Our findings suggest that collateral responses may be preserved across species. These findings may help extend the contribution of previous collateral network studies in the development of treatment strategies to address the problem of antibiotic resistance.

## Abstract (precis)

As drug resistance continues to grow, treatment strategies that turn resistance into a disadvantage for the organism will be increasingly relied upon to treat infections and to lower the rate of multidrug resistance. The majority of work in this area has investigated how resistance evolution toward a single antibiotic effects a specific organism’s collateral response to a wide variety of antibiotics. The results of these studies have been used to identify networks of drugs which can be used to drive resistance in a particular direction.

## INTRODUCTION

The discovery and introduction of antibiotics has been called the greatest health care advancement in history (1). In addition to reducing morbidity and mortality from bacterial infections, antibiotics have made modern medical procedures such as surgery, organ transplants, and chemotherapy possible (2). Unfortunately, the widespread use of these drugs has resulted in extensive resistance, which threatens to reverse many of the medical advancements made in the last century and return us to a preantibiotic era (3). A slowdown in the discovery and development of new antibiotics, due to scientific difficulties and financial and regulatory hurdles (4, 5), means that society cannot rely upon new antimicrobials becoming available. Given this critical junction, there is an urgent need to consider treatment strategies that combat resistance and prolong the use of our current antibiotic arsenal.

Treatment strategies that turn resistance into a disadvantage for the organism are the most desirable and are referred to as collateral sensitivity (CS)-informed antimicrobial strategies (6). CS is when resistance to one antibiotic simultaneously results in susceptibility to another (7). Over the last decade, several studies have considered how resistance evolution toward a single antibiotic effects CS and its converse, cross-resistance (CR), for a wide variety of antibiotics and bacterial species (8–20). The results of these works have been used to identify networks of drugs which can be used to drive resistance in a particular direction to take advantage of CS.

To date, most of this work has been performed using specific laboratory-adapted strains (8–12, 15, 17, 18, 20) or a limited number of clinical isolates (6, 13, 14, 16). While these studies have been vital to our understanding of CS in a wide variety of species, little remains known regarding the extent of evolutionary conservation or the universality of CS and CR across species (21). Such answers are important for determining the predictability of collateral responses. Recent work performed using several clinically resistant urinary tract Escherichia coli isolates found that collateral responses may be predictable across a species (6).

To investigate the extent of evolutionary conservation, we sought to identify commonalities in collateral networks of the ESKAPE pathogens—Enterococcus faecium, Staphylococcus aureus, Klebsiella pneumoniae, Acinetobacter baumannii, Pseudomonas aeruginosa, and Enterobacter cloacae (1). Members of the ESKAPE group are capable of causing a wide variety of illnesses, including bacteremia and skin, soft tissue, respiratory, and urinary tract infections (22–27). Our networks were derived from populations systematically evolved to five antimicrobials with diverse modes of action under uniform growth conditions. Whole-genome sequencing was used to characterize the molecular mechanisms involved in resistance and collateral responses. Our results suggest that collateral responses may be consistent across the species for select drugs.

## RESULTS

### Evolution leads to high resistance levels in both populations and isolates.

**FIG 1:**
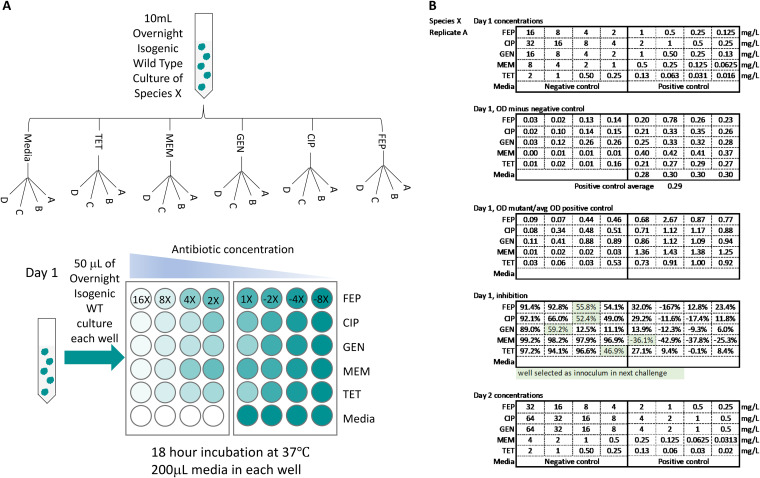
Resistance evolution of ESKAPE pathogens to individual drugs. An illustration of the initial evolution process and an example of the decision-making process used to determine which well to use for passage are shown. (A) An overnight culture of wild-type species X was used to inoculate microtiter plates containing different drugs with increasing concentrations or with medium only (Media). Each plate also included negative controls. Four replicate populations were created for each condition. The highest concentration where growth was present was used to inoculate the next concentration challenge. E. faecium, S. aureus, and K. pneumoniae were evolved for 18 days, while A. baumannii, P. aeruginosa, and E. cloacae were evolved for 12 days. (B) The top table shows day 1 drug concentrations. OD values of the evolved lineages minus the negative-control OD values are shown in the second table. OD values of the evolved mutants normalized by the OD value of the medium-only-evolved lineage are show in the third table. The fourth table shows the calculated percent inhibition for each well. Wells with the best growth and less than 60% inhibition were selected as the inoculum for the next resistance evolution challenge. These wells are highlighted in green. The bottom table lists concentrations used in the next challenge.

Wild-type (WT) strains of E. faecium, S. aureus strain Newman, K. pneumoniae, A. baumannii, P. aeruginosa, and E. cloacae were evolved in four replicate lineages, designated A, B, C, and D, to increasing concentrations of five broad-spectrum antibiotics (Table 1). To account for possible resistance phenotypes due to medium evolution, parallel medium-only evolution experiments were also performed using the WT for each species. These replicate lineages were designated MA, MB, MC, and MD. The evolution of each lineage was performed by growing a single freshly restreaked colony of each WT organism overnight in liquid medium (Fig. 1). An aliquot of each overnight culture was inoculated into five different antibiotic conditions with increasing antibiotic concentrations and grown for 18 h. All evolution experiments began at antibiotic concentrations eight times less than the corresponding WT MIC values (Table 2). At the end of each growth period, the optical density was measured and the most resistant culture from each replicate with an inhibition less than 60% (see Materials and Methods) was used as the next inoculum. E. faecium, S. aureus, and K. pneumoniae were evolved for 18 days, while A. baumannii, P. aeruginosa, and E. cloacae were evolved for 12 days. A total of 144 lineages (120 drug evolved and 24 medium only) were evolved.

**TABLE 1:** List of antibiotics used in this study

Antibiotic	Abbreviation	Class (subclass)	Target
Cefepime	FEP	Beta-lactam (cephalosporin)	Cell wall synthesis
Ciprofloxacin	CIP	Fluoroquinolone	DNA gyrase
Gentamicin	GEN	Aminoglycoside	Protein synthesis, 30S
Meropenem	MEM	Beta-lactam (carbapenem)	Cell wall synthesis
Tetracycline	TET	Tetracycline	Protein synthesis, 30S

**TABLE 2:** Starting concentrations for adaptation experiments

Antibiotic	Starting concn (mg/liter)					
	E. faecium	S. aureus	K. pneumoniae	A. baumannii	P. aeruginosa	E. cloacae
FEP	0.25	0.125	0.002	0.125	0.125	0.0156
CIP	0.25	0.0039	0.001	0.0039	0.0039	0.002
GEN	0.0313	0.0156	0.0039	0.0313	0.0625	0.0625
MEM	0.25	0.0039	0.002	0.0039	0.0625	0.0156
TET	0.0039	0.0039	0.0078	0.0156	0.5	0.125

**FIG 2:**
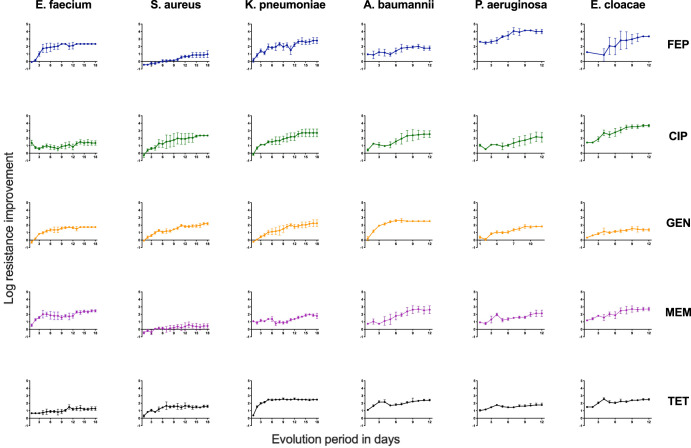
Change in drug tolerance over the course of the resistance evolution period. Each graph shows the resistance improvement, the concentration tolerated by the isolate relative to the corresponding WT IC_90_ value, over the course of the resistance evolution period. All normalized values were log transformed. Drugs used in the experiments are color coded and indicated on the right, while species investigated are indicated at the top. Each graph is comprised of three to four replicates. Each point reflects the mean resistance improvement of the isolates on a specific day. Error bars represent a single standard deviation. E. faecium, S. aureus, and K. pneumoniae were evolved for 18 days, while A. baumannii, P. aeruginosa, and E. cloacae were evolved for 12 days.

Overall, resistance increased steadily within each resistance group (Fig. 2). A resistance group refers to all species evolved to the same antibiotic (6); for example, ESKAPE species evolved to cefepime (FEP) are the FEP resistance group. Resistance improvement values of less than 1 were occasionally observed in the early days of the evolution period and indicated that some populations were less resistant than initially determined. The first day’s resistance improvement of P. aeruginosa PAO1 to FEP was markedly higher than that of any other species and drug combination. This large gain in resistance improvement was likely the result of the inoculum effect, which is frequently observed in *in vitro* studies using cephalosporins (28). Plateaus in the evolution process were observed in every resistance group. Nearly all populations (94%) completed the evolution period with at least a 10-fold increase in resistance compared to their corresponding WT. E. cloacae populations evolved to ciprofloxacin (CIP) and cefepime were able to grow in concentrations 1,000-fold greater than their WT (Fig. 2). In contrast, S. aureus populations evolved to meropenem had the smallest increase, 5-fold or less, in resistance relative to their WT.

Following resistance evolution, four isolates from each of the most evolved populations were tested for their individual resistance levels. The isolate results were then compared with the final resistance evolution concentrations of their corresponding populations. To determine if the selected medium influenced the resistance evolution of each species, isolates from all medium-evolved lineages were also tested for their individual resistance levels.

**FIG 3:**
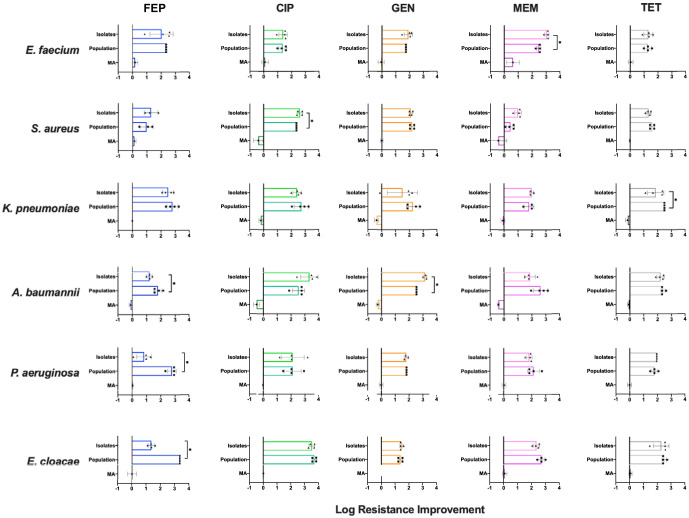
Resistance improvement of drug-evolved isolates relative to their corresponding populations. Four biological isolates from each of the evolved populations were evaluated to determine the extent of their individual resistances. These values were converted to resistance improvement by normalization to their corresponding WT IC_90_ values and then compared to resistance improvement of the population. The final resistance evolution concentration of the corresponding population was used to generate a resistance improvement value. Isolates from all medium-evolved lineages had their IC_90_ values tested to determine how the medium chosen may have affected resistance evolution of each species. All normalized values were log transformed, and error bars represent a single standard deviation. The bars at the top of each graph represent the resistance improvement of the isolates. The middle bars show the improvement of the evolved populations. The bars at the bottom of each graph show the resistance improvement of the media-evolved lineages (media only [MA]). If no MA bar is visible, it is because the IC_90_ value is the same as that of the WT. The resistance improvement of the isolates closely mirrored that of their corresponding populations. An asterisk denotes a statistically significant difference between the resistance improvement of the population and isolates.

In general, the resistance levels of the isolates mirrored those of their respective populations (Fig. 3). However, there were seven instances where the differences in the population and isolate resistance levels were statistically significant. Approximately half of these cases involved lineages evolved to the beta-lactams and in particular those evolved to cefepime (Fig. 3). These isolates were markedly less resistant than their corresponding populations. We speculate that this difference may be due to storage of our populations at −80°C prior to the performance of subsequent phenotypic testing. Loss of resistance following cryostorage has previously been observed in Helicobacter pylori resistant to amoxicillin (29) and S. aureus resistant to methicillin (30, 31) and vancomycin (32). Alternatively, the difference may be due to the inoculum effect, which is known to occur with beta-lactams, in particular cephalosporins (28, 33). In the case of strain PAO, the inoculum effect is most likely the cause of the difference observed between the populations and their corresponding isolates.

With the exception of the meropenem resistance group, the medium used in the evolution experiments does not appear to have promoted or hindered evolution (Fig. 3). E. faecium, S. aureus, and A. baumannii MA lineages all experienced notable changes in their susceptibility to meropenem. Interestingly, the medium used appears to have had a slightly negative impact on the resistance evolution of A. baumannii.

### Universal cross-resistance relationships emerge across the species.

We investigated the collateral responses of laboratory strains of E. faecium, S. aureus, K. pneumoniae, A. baumannii, P. aeruginosa, and E. cloacae after resistance evolution. Following the evolution period, four isolates from each of the most evolved populations were tested against all five drugs—cefepime (FEP), ciprofloxacin (CIP), gentamicin (GEN), meropenem (MEM), and tetracycline (TET)—used in this study. A total of 480 isolates were inoculated into the different antibiotic conditions and allowed to grow for 18 h. The 90% inhibitory concentration (IC_90_) values of the resistant isolates were compared with those of their corresponding WTs for the different antibiotics.

**FIG 4:**
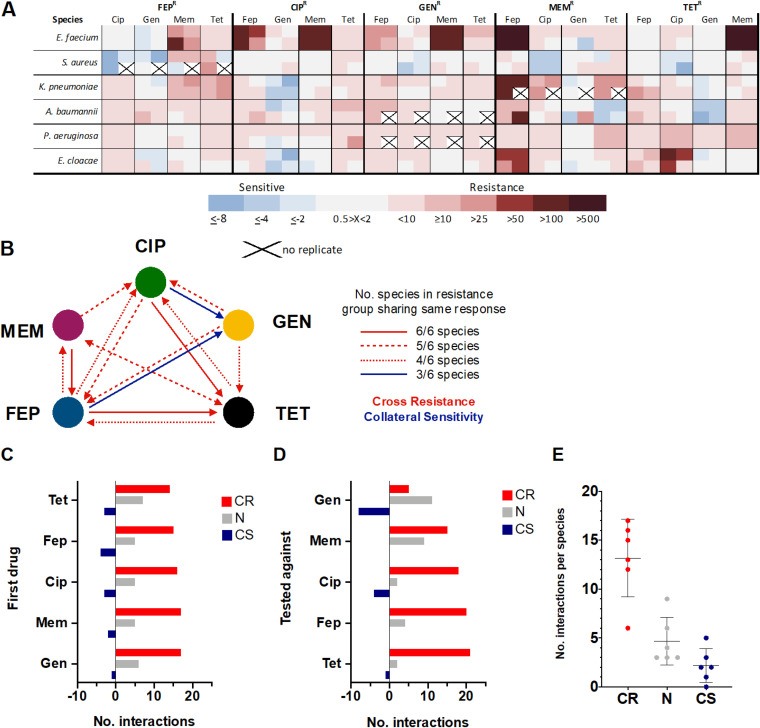
Collateral interactions resulting from resistance evolution to a single antibiotic. Isolates from the most evolved lineages were tested against the other antibiotics used in this study. (A) Heat map results are grouped according to resistance group. Rows identify the species tested, while the challenge drugs are listed by column. Each individual square represents the results of a single lineage. A collateral response was decided for each species of a resistance group when ≥ 50% the species’ lineages exhibited resistance or sensitivity. Blue indicates CS, while red represents CR. (B) A high degree of cross-resistance agreement was observed among the different species, with a total of 14 relationships being found. The robustness of the relationship is conveyed by the line style. Solid red lines reflect all species of a resistance group sharing the same response. Dashed red lines represent responses where five out of six species shared the same response. Dotted red lines show responses where four of the six species shared the same response. Two global CS relationships were found and are shown as blue lines. CS relationships were less robust, with only half the species of the resistance group exhibiting the response. (C and D) The number of collateral interactions as a function of the drug to which resistance evolved (C) and the drug against which the species was tested (D). (E) Average number of collateral interactions per species.

Overall, collateral responses were widespread and observed in 55% (254/464) of possible instances. Collateral responses were proportionately distributed across all drugs and species (Fig. 4A). Cross-resistance accounted for 80% (202/254) of all the collateral responses observed. Occurrences of collateral sensitivity were less frequent and comprised 20% (52/254) of the observed collateral responses. Cross-resistance or collateral sensitivity was defined as a 2-fold increase or decrease in the MIC of the antibiotic-evolved strain relative to its WT (9).

A collateral response was considered universal when all species of a resistance group shared the same response toward a particular drug. A collateral response was considered broad when five out of six species of a resistance group shared the same response toward the same drug. Finally, a collateral response was considered extended when four out of six species of a resistance group shared the same collateral response toward the same drug. A collateral response was decided for each species of a resistance group when ≥ 50% of the lineages exhibited resistance or sensitivity.

A review of the cross-resistance instances revealed a high degree of agreement among the different species, with three universal, six broad, and five extended cross-resistance relationships being found (Fig. 4B). The universal relationships observed were cefepime evolution resulting in tetracycline cross-resistance, ciprofloxacin evolution conferring cross-resistance to tetracycline, and meropenem evolution resulting in cefepime cross-resistance. The broad relationships found were cefepime cross-resistance in lineages evolved to ciprofloxacin or gentamicin, ciprofloxacin cross-resistance in lineages evolved to gentamicin and meropenem, and finally bidirectional cross-resistance between tetracycline and meropenem. The extended relationships observed were cefepime resistance resulting in cross-resistance to ciprofloxacin and meropenem, tetracycline resistance conferring cross-resistance to ciprofloxacin and cefepime, and cross-resistance to tetracycline resulting from gentamicin resistance. Conspicuously absent is cross-resistance to gentamicin. Surprisingly, resistance evolution of E. faecium toward any single drug invariably resulted in cross-resistance toward meropenem (Fig. 4A).

Global collateral sensitivity responses were fewer and less robust (Fig. 4B). Gentamicin collateral sensitivity was observed in half of the resistance groups evolved to cefepime or ciprofloxacin. Interestingly, resistance evolution of S. aureus toward any single drug consistently resulted in collateral sensitivity toward ciprofloxacin (Fig. 4A).

Review of the collateral response data based on the drug to which it had evolved revealed an evenness in the number of cross-resistance and collateral sensitivity interactions observed across the drugs used in the evolution process (Fig. 4C). Evaluating these same data on the basis of per drug tested to revealed an inequality in the distribution of cross-resistance across the tested drugs (Fig. 4D). All resistance groups tested against tetracycline or cefepime were frequently observed to be cross-resistant to these two drugs (20 and 21 instances, respectively), indicating that these two drugs would likely be a poor second drug choice in a drug cycling plan. In contrast, resistance groups tested against gentamicin consistently displayed either a neutral response (11 instances) or a collaterally sensitive response (eight instances) to this drug, indicating that gentamicin might be good second drug choice in a multistep treatment plan. Collateral sensitivity to gentamicin following resistance evolution has previously been observed in several single-species adaptive evolution studies (6, 16, 34). Analysis of the response data on a per species basis uncovered an average of 13 ± 4 (standard deviation [SD]) cross-resistance responses, 5 ± 2 neutral responses, and 2 ± 2 collateral sensitivity responses per species. Deviating from these values considerably were the S. aureus populations, which had only six cross-resistance interactions but nine neutral and five collateral sensitivity interactions (Fig. 4E).

### Evolutionary responses are divergent or conserved in a drug-dependent manner.

To explore the molecular basis of the drug resistances observed in our experiments, we sequenced a single isolate from each of the most evolved populations (120 drug-evolved isolates and 24 medium only evolved) and a WT ancestral isolate for each species. The sequenced isolates were then analyzed according to their resistance group. Mutations arising from evolution to the medium were identified and removed from further analysis in the drug-evolved isolates.

**FIG 5:**
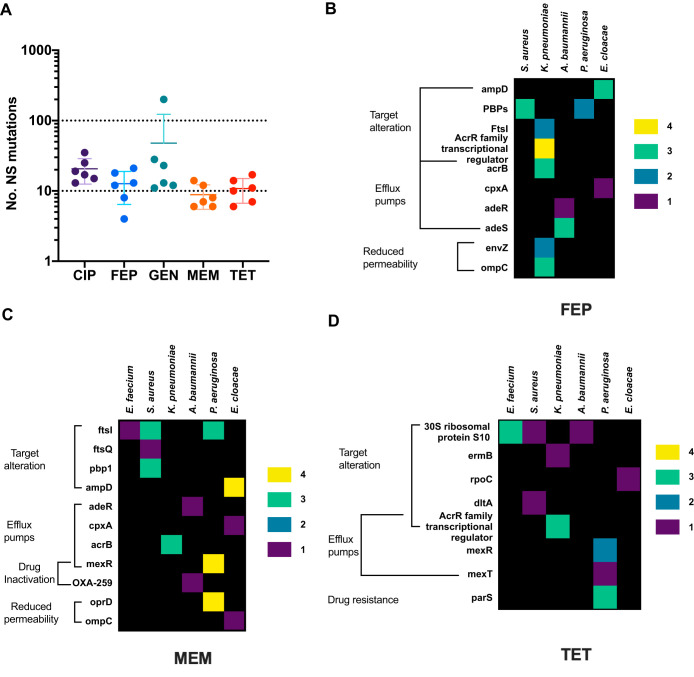
Beta-lactams and tetracycline provoke divergent genomic responses. Single isolates from each of the most evolved populations were sequenced and compared to the ancestral WT and medium-evolved lineages to identify mutations that arose from resistance evolution. (A) The average number of nonsilent (NS) mutations per drug. (B to D) NS mutations observed in lineages with resistance evolution to cefepime (B), meropenem (C), and tetracycline (D). The mutations shown were found in genes associated with resistance determinants as defined by the Comprehensive Antibiotic Resistance Database (CARD) (71). Columns detail the species, while rows indicate genes in which mutations were observed. Genes are grouped by resistance mechanism. Different colors represent the number of the lineages carrying a mutation in the listed gene per species, ranging from 1 to 4. A black background indicates an absence of gene mutation. E. faecium lineages evolved to cefepime did not include mutations in genes associated with resistance determinants defined by CARD, and therefore this species is not included in panel B.

A total of 874 mutations was identified in the resistant isolates, and of these, 69% (605/874) were nonsynonymous (NS) mutations. The average number of NS mutations per drug was less than 20, with the exception of that for gentamicin, where a single hypermutating E. faecium isolate had 187 NS mutations (Fig. 5A). Approximately 26% (155/605) of NS mutations were located in genes previously associated with resistance.

Analysis of the mutations revealed two categories of genomic response—a diverse response group and a conserved one (see Data Sets S1 to S5 in the supplemental material). The diverse response group was comprised of the beta-lactam and tetracycline resistance groups and was characterized by changes in a variety of pathways (Fig. 5B to D; Data Sets S1 to S3). For example, resistance groups evolved to meropenem had mutations in genes related to target modification, efflux pumps, beta-lactamases, and reduced permeability (Fig. 5C; Data Set S2). E. faecium, S. aureus, and P. aeruginosa lineages all had target modification mutations, while in K. pneumoniae, A. baumannii, and E. cloacae lineages, mutations were found in efflux pumps. P. aeruginosa lineages also had mutations in efflux pumps and a gene related to reduced permeability. The genetic response to the beta-lactams appears to have been preserved within certain species. For example, E. cloacae lineages evolved to both cefepime and meropenem had mutations in *ampD* and *cpxA* (Data Sets S1 and S2). Similar findings were observed in A. baumannii and K. pneumoniae lineages. Interestingly, E. faecium lineages evolved to FEP and MEM possessed few mutations, which could be associated with conferring beta-lactam resistance. A closer examination of the E. faecium WT species revealed genes related to cell envelope stress response and to altering cell wall charge but none associated with beta-lactam resistance.

**FIG 6:**
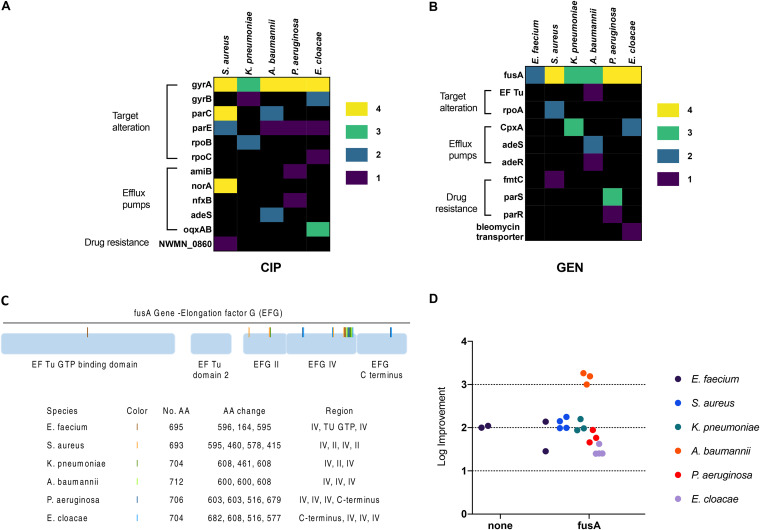
Resistance evolution to gentamicin is characterized by localized mutations in the *fusA* gene. Single isolates from each of the most evolved populations were sequenced and compared to the ancestral WT and medium-evolved lineages to identify mutations that arose from resistance evolution. (A and B) NS mutations were observed in lineages evolved to ciprofloxacin (A) and gentamicin (B). The mutations shown were found in genes associated with resistance determinants as defined by the Comprehensive Antibiotic Resistance Database (CARD) (71). Columns detail the species, while rows indicate genes in which mutations were observed. Genes are grouped by resistance mechanism. Different colors represent the number of the lineages carrying a mutation in the listed gene per species, ranging from 1 to 4. A black background indicates an absence of gene mutation. (C) Schematic of the *fusA* gene highlighting the mutations that arose during resistance evolution. The table below the gene schematic lists the species in which the mutations are observed, provides a color code for each species, supplies the number of amino acids (AA) present in the gene, identifies the amino acids changed, and supplies information about the gene region in which the amino acid change is located. The majority of mutations in *fusA* were found between AA516 and AA608 (numbered according to strain PAO1) in domain IV of the gene. Domain IV is home to two highly conserved loops where aminoglycosides are known to bind. (D) A mutation in the *fusA* gene was associated with at least a 100-fold improvement in IC_90_ relative to that of the WT for all species expect E. faecium. A. baumannii isolates with *fusA* mutations had 1,000-fold improvements.

In contrast, the conserved response group was composed of resistance groups evolved to drugs (ciprofloxacin and gentamicin) that elicited a limited number of changes in select pathways (Fig. 6A and B; Data Sets S4 and S5). Indeed, all species evolved to gentamicin had mutations in the *fusA* gene. Analysis of the *fusA* gene from all our evolved isolates revealed that the observed mutations were confined primarily to domain IV. This domain is situated at the A site of the 30S subunit and interacts with mRNA, tRNA, and the decoding center on h44 (35). Mutations in this domain have previously been identified as responsible for kanamycin resistance (36). Alignment of the *fusA* gene from all six species revealed that 80% of the domain IV mutations were contained between amino acids (AA) 510 and 610 (based on strain PAO1 numbering) (Fig. 6C). AA516, AA595, and AA608 were mutated in more than one species. AA608, in particular, was mutated twice in K. pneumoniae and once each in A. baumannii and E. cloacae. Previous work conducted using PAO1 hypothesized that *fusA* mutations led to structural changes that altered the binding of aminoglycosides to h44, thus lowering their affinity for the ribosome (37). *fusA* mutations have previously been shown to confer resistance to aminoglycosides in S. aureus (38, 39), P. aeruginosa (40, 41), and E. coli (11, 42).

In general, mutations in the *fusA* gene correlated with a 100-fold increase in resistance to gentamicin relative to the WT strains (Fig. 6D). The exceptions to this observation were the A. baumannii lineages, which had a 1,000-fold increase in resistance, and the E. faecium lineages, which had a 100-fold increase in resistance regardless of mutations in the *fusA* gene. The A. baumannii lineages had additional mutations in the two-component system AdeRS. This system regulates the resistance-nodulation-cell division (RND) pump AdeABC, which is known to confer high-level resistance to aminoglycosides, beta-lactams, fluoroquinolones, tetracyclines, and several other antimicrobials (43). The WT E. faecium strain used possessed a wide array of antibiotic efflux mechanisms. It is likely that the gentamicin resistance observed in those lineages without *fusA* mutations is due to these mechanisms.

In addition to *fusA* mutations, the Gram-negative species had additional mutations in efflux pump regulators, in *parRS* (P. aeruginosa), and in the two-component envelope stress response system, *cpx* (K. pneumoniae and E. cloacae). Mutations in *cpxA* are known to confer aminoglycoside resistance in E. coli (11, 44, 45). The presence of *cpx* mutations in E. cloacae and K. pneumoniae lineages may explain the observed cross-resistance to cefepime and meropenem (K. pneumoniae only).

Another example of conserved mutations was observed in lineages evolved to ciprofloxacin. With the exception of E. faecium, all species had mutations in *gyrA* or *gyrB* (DNA gyrase) and *parC* or *parE* (topoisomerase IV), the primary targets of quinolone drugs. The majority of mutations observed in our study were located in the quinolone resistance-determining region (46, 47) at two specific amino acid sites (AA90 or AA94, numbered according to S. aureus). The genome of the E. faecium WT was found to contain the *pmrA* gene, which is known to confer low-level ciprofloxacin resistance (48–50). It is possible that the *pmrA* gene is responsible for the observed resistance and may explain the absence of drug target gene mutations in these lineages.

Previous work has shown that large deletions are commonly observed in laboratory evolution studies (51). Large deletions were found in every species and under every drug condition investigated. While the majority of deleted genes were classified as hypothetical proteins with unknown functions, there were a few instances where the gene deletions were either extensive or included known resistance functions. For example, the tetracycline-evolved P. aeruginosa isolates all had several large (>10) gene cluster losses. In one isolate in particular, these deletions included the *mexR*, *mexA*, *mexB*, and *oprM* genes, which are known to be involved with drug resistance (52) (Data Set S3). Since neither the mutations nor the gene deletions were reintroduced into their corresponding WT organisms, it is not possible to assess their contributions to the observed phenotypic response.

### Genetic basis for observed collateral sensitivity is obscure.

We sought to provide a genomic explanation for the observed collateral sensitivity in resistance groups evolved to FEP and CIP. Using gene ontology (GO) as a means of categorizing the observed mutations, we examined the difference between lineages with and without GEN collateral sensitivity within the same resistance group and then across resistance groups. GO provides information about the molecular function, cellular component, and biological process each gene product may carry out (53, 54). The GO characterization of each mutated gene was found in the UniProt Knowledgebase (UniProtKB) (55). Information pertaining to molecular function (MF) was the most consistently available, and thus MF terms were used to categorize the mutation data. MF terms describe the molecular activities of individual gene products. Genes for which a product has not been annotated are described as having unknown activities.

In FEP-evolved lineages with GEN collateral sensitivity, genes with unknown activities comprised the largest portion of mutations (31%), followed by those associated with ATP binding (17%) and DNA binding (12%). FEP-evolved lineages without collateral sensitivity had a similar distribution of mutations. Genes with unknown activities accounted for one-third of the mutations, followed by those associated with ATP binding (18%) and DNA binding (8%). Approximately 33% of all the mutations observed in the nonsensitive lineages were also found to be present in those with sensitivity.

Analysis of CIP-evolved lineages with and without GEN collateral sensitivity yielded similar results. In the collaterally sensitive lineages, genes with unknown activities once again comprised the largest group of mutations (41%), followed by those associated with ATP binding (32%) and DNA binding (14%). In the nonsensitive lineages, genes with unknown activities represented the largest category of mutations (38%), followed by those associated with ATP binding (31%). Genes with products related to DNA binding accounted for only 5% of the observed mutations. Analogous to the FEP-evolved lineages, 37% of mutations found in the nonsensitive lineages were also found in the sensitive lineages.

The large fraction of genes with unknown activities found in all lineages in both resistance groups obscure the genetic mechanisms responsible for the observed collateral sensitivity. Additionally, the mutation overlap observed between lineages with and without collateral sensitivity within each resistance group makes it unclear as to why some lineages became sensitive and others did not. These similarities prevent conclusions being drawn across resistance groups.

## DISCUSSION

To date, the bulk of collateral network studies have considered only the effects of resistance evolution of a single laboratory species. Questions remain about the universality of these findings (21). Recent studies have attempted to extend these findings by comparing the results of laboratory strains and clinical isolates (16), by considering the conservation of collateral findings across different clinical isolates of the same species (6), and by investigating the role that genetic background plays in collateral interactions (21). What has not yet been explored is the extent of conservation of collateral networks across different species. The work presented here sought to address this question.

To this end, we performed a systematic resistance evolution study of the ESKAPE pathogens under uniform growth conditions using five clinically relevant antibiotics with diverse modes of action. Our evolved lineages were analyzed for collateral effects and the molecular mechanisms behind the observed phenotypes.

A total of 14 universal CR and two global CS relationships were observed. Similar to previous studies (6, 8–10), the magnitude of collateral interactions observed was generally low (<10-fold) (Fig. 4A). In contrast to an earlier study that considered several clinical isolates of the same species (6), the collateral responses observed in our work did not vary statistically across the response groups (Fig. 4C). Instead, collateral responses varied by the drug tested against and the species (Fig. 3E and 4D). Regardless of the antimicrobial to which a lineage had evolved, if the next drug exposure was to gentamicin, there was a greater likelihood for a CS interaction to take place than a CR interaction. Conversely, if the next drug exposure was to tetracycline or cefepime, the likelihood of a CR interaction was high. Our evolved S. aureus lineages were recalcitrant to collateral effects, whereas PAO1 became multidrug resistant following resistance evolution toward one antimicrobial. This difference is likely due to PAO1 having substantially more inherent resistance mechanisms than the S. aureus strain used.

Drug-dependent divergent and conserved evolutionary trajectories were revealed in genomic analyses of the most evolved lineages. Cefepime, meropenem, and tetracycline elicited a diverse genomic response that involved mutations in a wide range of genes across the different species, whereas for ciprofloxacin and gentamicin, the genomic response appeared to be limited to a few genes across the different species. In the case of gentamicin, mutations in the *fusA* gene could be correlated with an increase of 100-fold resistance improvement in most species. The observed diverse and converse genomic response could not be correlated with occurrence of CR or CS (Fig. 4C).

To date, there are several resistance evolution studies of individual members of the ESKAPE group (9, 13, 16, 56–61); however, no study has examined the commonalities that arise from resistance evolution of these species under uniform conditions toward more than one class of antibiotic. A study by Michiels et al. considered the persistence of the ESKAPE pathogens toward aminoglycosides but was performed under inconsistent environmental conditions (62). The work presented in this study is the first to consider the simultaneous resistance evolution of all ESKAPE species toward a broad suite of antibiotics under uniform conditions and to identify commonalities in their collateral and genomic responses.

The findings presented here, while addressing an existing shortcoming, do have limitations. First, our results stem from a laboratory evolution environment in which conditions, such as the absence of competition for nutrients, environmental stresses, etc., are unlike those encountered in a clinical setting. Nevertheless, some of the mutations observed in our work have also been found in clinical isolates. In particular, gyrase mutations contributing to ciprofloxacin resistance have been previously observed in clinical isolates of E. coli (63), Staphylococcus epidermidis (64), S. aureus (65), A. baumannii (66), and P. aeruginosa (67). Additionally, the work presented here does not consider the role of horizontal gene transfer (HGT) in multidrug resistance. HGT is an important driver of antibiotic resistance for several of the ESKAPE pathogens, in particular those from the *Enterobacteriaceae* and *Enterococcaceae* families (68). It is important to note that the choice of adaptive evolution methodology plays a role in the evolutionary trajectory taken, in particular, the roles that biofilms (61, 69) and population size (70) play in the outcomes of resistance evolution. Moreover, the mutations observed in this work were not reintroduced back into the species where they were found, and therefore, it cannot conclusively be said that they confer the resistance attributed to them. Further, the number of drugs used in our study was small compared with other laboratory resistance evolution studies (6, 8–11, 17, 38, 51). This was due to difficulty in finding antimicrobials to which all our species were sensitive. It is likely that we could have included more antimicrobials following the resistance evolution period. Finally, our work considers only a single laboratory representative of each ESKAPE member. The results of this work would be strengthened by a larger study that included a larger number and wider variety of strains from each ESKAPE member. Despite the aforementioned limitations, the results of our work serve as a first attempt in addressing the question of universality of collateral interactions across a wide range of species.

## MATERIALS AND METHODS

### Bacteria and reagents.

Drug-sensitive E. faecium (DSM 2146), S. aureus strain Newman, K. pneumoniae (DSM 30104), A. baumannii (ATCC 17978), P. aeruginosa (PAO1), and E. cloacae (ATCC 13047) were evolved to five antibiotics: cefepime, ciprofloxacin, gentamicin, meropenem, and tetracycline. Drug stock solutions were prepared every 10 days and stored at −20°C until needed. Working stock solutions were prepared from frozen stocks every 4 days and stored at 4°C. All experiments were performed in cation-adjusted Mueller-Hinton broth (Sigma) supplemented with 0.5% (wt/vol) glucose.

### Antimicrobial susceptibility testing.

Wild-type (WT) 90% inhibitory concentrations (IC_90_) and MICs were established for every organism to each antibiotic. Each WT organism was plated on nonselective medium and allowed to grow overnight. Four individual colonies from each species were then randomly selected and grown in nonselective medium for 4 to 6 h. After the allotted growth period, the optical density (OD) of each preculture was measured and a cell concentration was determined. Dilutions of approximately 10^3^ cells/μl were made for all precultures.

All susceptibility tests were performed in 96-well microtiter plates containing 150 μl of medium. A 10-dilution-step 2-fold drug gradient was used on every microtiter plate. Each plate also included positive (non-antibiotic-containing medium plus bacterial inoculum) and negative (non-antibiotic-containing medium only) controls. All inoculated wells received 10 μl of diluted preculture. Following inoculation, the plates were incubated at 37°C without shaking for 18 h. After the allotted growth period, the OD at 600 nm (OD_600_) of each plate was measured using an Epoch absorbance reader (BioTek). The average OD_600_ values of the negative controls were subtracted from all remaining OD_600_ values. The percent inhibition was calculated according to equation 1:

(1) $$\text{Percent inhibition}=1-\frac{{\text{OD}}_{600\text{, growth}}-{\text{OD}}_{600,\text{negative control}}{}_{}}{{\text{OD}}_{600,}{}_{\text{positive control}}-{\text{OD}}_{600,}{}_{\text{negative control}}}$$

The drug concentration causing 90% growth inhibition was determined using a Python script.

### Evolution of antibiotic-resistant isolates.

All evolution experiments began at concentrations eight times lower than the corresponding WT MIC and increased in 2-fold steps. All evolution experiments were performed in quadruplicate in two 24-well microtiter plates. Each well contained 2 ml of medium. Positive (non-antibiotic-containing medium plus bacterial inoculum) and negative (non-antibiotic-containing medium only) controls were included in all evolution experiments. The positive-control isolates are referred to as medium-adapted (MA) lineages.

A single WT colony of each species was grown overnight in liquid medium. This overnight culture was used as the inoculum to begin the evolution experiments. All inoculated wells received 50 μl of overnight culture. Following inoculation, the microtiter plates were incubated at 37°C without shaking for 18 h. After the allotted growth period, the OD_600_ of each plate was measured using an Epoch absorbance plate reader (BioTek) at a wavelength of 600 nm. The OD_600_ values of the negative control was subtracted from all remaining OD_600_ values. Percent inhibition was calculated according to equation 1. A 60% inhibition cutoff was used to determine the starting concentration of the next experiment. This concentration was referred to as the experimental MIC. The 60% inhibition value was selected based on previous work (38). The well with the best growth closest to the experimental MIC was used as the inoculum for the next evolution experiment. The evolution process was repeated for 12 (A. baumannii, P. aeruginosa, and E. cloacae) or 18 (E. faecium, S. aureus, and K. pneumoniae) days. Aliquots of the selected wells were frozen in 96-well plates every 3 days. Medium-only-evolved populations experienced no inhibition and were passaged daily. Aliquots of the medium-evolved populations were also frozen every 3 days. On the final day of evolution, aliquots from the most resistant wells that met the 60% inhibition cutoff were plated and grown overnight. A single colony from each agar plate was randomly picked and grown overnight in liquid medium. Aliquots of these overnight cultures were saved for future analysis. A total of 144 isolates were saved.

### Antimicrobial susceptibility testing postevolution.

Following resistance evolution, isolates of the drug-evolved and MA populations were profiled for their individual resistance to each antibiotic. Final exposure populations were plated on nonselective medium and allowed to grow overnight. Four colonies from each population were randomly selected and grown in nonselective medium for 4 to 6 h. These isolates were then tested against all five drugs used in the resistance evolution experiments. Susceptibility testing and data analysis were performed according to the method described in “Antimicrobial susceptibility testing.” Each WT species also had its antimicrobial susceptibility reestablished. MA and WT isolates were tested on the same day.

### Whole-genome sequencing.

A single isolated colony from each of the 144 evolved lineages and the six WT species was grown overnight in nonselective medium. Genomic DNA was isolated from the overnight cultures using an UltraClean microbial DNA isolate kit (MoBio Laboratories, Inc.). The DNA was sent to Macrogen for library construction using the TruSeq Nano (550 bp) DNA library prep kit (Illumina) and sequencing by Illumina MiSeq 300-bp paired end. Additional sequencing was performed by the Sequencing Core Facility at the Novo Nordisk Foundation Center for Biosustainability.

### Analysis of genome sequences.

The following chromosomes were used to align their corresponding sequence data: A. baumannii (GenBank accession no. CP018664, version CP018664.1), P. aeruginosa (accession no. NC_002516, version NC_002516.2), E. cloacae (accession no. NC_014121, NC_014108, and NC_014107; version NC_014121.1, NC_014108.1, and NC_014107.1), S. aureus strain Newman (accession no. NC_009641, version NC_009641.1). Reference genomes were unavailable for the specific E. faecium and K. pneumoniae strains used. The WT genomes of these species were *de novo* assembled using the De Novo assembly function in Genomics Workbench 9.0.1 (CLC Bio, Qiagen), annotated using RAST, and then used as a reference.

A minimum coverage of 30-fold was obtained for each isolate. All sequences were trimmed to remove low-quality reads and reads of less than 75 bp. All sequences were aligned to their corresponding reference genome. Point mutations and short indels were identified using the Quality-based variant detection option in Genomic Workbench 9.0.1. Only loci with a phred score of at least 30 and occurring with a frequency of at least 80% were used for analysis. Single nucleotide polymorphisms (SNPs) present in the WT in the medium-evolved lineages, or in at least two mutants resistant to different antibiotics from the same strain background, were excluded from further analysis, as they were considered to be present in the starting culture prior to the start of the experiments.

### Detection of large deletions.

Large deletions were identified in Genomic Workbench 9.0.1 using a previously described workflow (51). Briefly, all genomes were analyzed for large deletions (>300 nucleotides [nt]) using Genomic Workbench. Each genome was assembled *de novo*, and this assembly was then used as a reference on which to map the WT genome reads. Any WT reads that could not be mapped to the *de novo* reference were collected and *de novo* assembled into contigs. The newly assembled unmapped WT reads were considered to contain deleted regions. Only regions with more than 300 nt and greater than 30-fold coverage were used for further analysis.

## Supplementary Material

Supplemental file 1

Supplemental file 2

Supplemental file 3

Supplemental file 4

Supplemental file 5
